# Organogenesis during budding and lophophoral morphology of *Hislopia malayensis *Annandale, 1916 (Bryozoa, Ctenostomata)

**DOI:** 10.1186/1471-213X-11-23

**Published:** 2011-04-18

**Authors:** Thomas Schwaha, Timothy S Wood

**Affiliations:** 1 University of Vienna, Department of Morphology, Althanstraße 14, 1090 Vienna, Austria; 2 Wright State University, Department of Biological Sciences, 3640 Colonel Glenn Highway, Dayton, OH 45435 USA

## Abstract

**Background:**

Bryozoans represent a large lophotrochozoan phylum with controversially discussed phylogenetic position and in group relationships. Developmental processes during the budding of bryozoans are in need for revision. Just recently a study on a phylactolaemate bryozoan gave a comprehensive basis for further comparisons among bryozoans. The aim of this study is to gain more insight into developmental patterns during polypide formation in the budding process of bryozoans. Particular focus is laid upon the lophophore, also its condition in adults. For this purpose we studied organogenesis during budding and lophophoral morphology of the ctenostome bryozoan *Hislopia malayensis*.

**Results:**

Polypide buds develop on the frontal side of the developing cystid as proliferation of the epidermal and peritoneal layer. Early buds develop a lumen bordered by the inner budding layer resulting in the shape of a two-layered sac or vesicle. The hind- and midgut anlagen are first to develop as outpocketing of the prospective anal area. These grow towards the prospective mouth area where a comparatively small invagination marks the formation of the foregut. In between the prospective mouth and anus the ganglion develops as an invagination protruding in between the developing gut loop. Lophophore development starts with two lateral ridges which form tentacles very early. At the lophophoral base, intertentacular pits, previously unknown for ctenostomes, develop. The ganglion develops a circum-oral nerve ring from which the tentacle nerves branch off in adult zooids. Tentacles are innervated by medio-frontal nerves arising directly from the nerve ring, and medio-frontal and abfrontal nerves which originate both from an intertentacular fork.

**Conclusions:**

We are able to show distinct similarities among bryozoans in the formation of the different organ systems: a two-layered vesicle-like early bud, the ganglion forming as an invagination of the epidermal layer in between the prospective mouth and anal area, the digestive tract mainly forming as an outpocketing of the prospective anal area, and the lophophore forming from two lateral anlagen that first fuse on the oral and afterwards on the anal side. Future studies will concentrate on cyclostome budding to complement our knowledge on developmental patterns of bryozoans.

## Background

The Bryozoa represent a large lophotrochozoan phylum consisting of sessile filter-feeders comprising over 6000 extant species. The phylum consists of three large clades: The Phylactolaemata, the Stenolaemata and the Gymnolaemata (Ctenostomata and Cheilostomata) [1]. The relationship in between the different clades and also to other phyla remains controversially discussed [2]. The Phylactolaemata represent a small group of freshwater inhabiting species. From a phylogenetic perspective they are interesting, since they are often regarded as the most basal bryozoans and show several morphological characters that distinguish them from all remaining bryozoans, such as an epistome and body wall musculature [1,3]. In particular their sexual development, however, is heavily altered, probably as an adaptation to freshwater habitats, and therefore impedes comparisons to other phyla and bryozoans. Within the Gymnolaemata, the Ctenostomata are a group of uncalcified, comparatively simple species that are currently regarded as paraphyletic with the species-richest bryozoan group Cheilostomata as well as Cyclostomata being ingroups [4-7]. Consequently, they represent an important clade for addressing phylogenetic questions of bryozoans.

As previously mentioned by Nielsen [8], budding in bryozoans, in particular organogenesis, is only poorly known. Schwaha et al. [9] recently studied the organogenesis in the budding process of the phylactolaemate *Cristatella mucedo *and established a first comprehensive study for further comparative purposes. Detailed investigations on the polypide development during the budding process of ctenostome bryozoans were only carried out by Davenport [10] for *Paludicella articulata*. Soule [11] studied several species, but only gave generalized and short descriptions with poor documentation. Accordingly, ctenostome budding requires new data to gain more insight into general trends and patterns in bryozoan budding. This study focuses on the organogenesis in the budding process of the ctenostome *Hislopia malayensis*, a species occurring in freshwater habitats of South East Asia [12]. Since it shows many ancestral traits among ctenostomes [13], it represents a suitable species for the current study. With the lophophoral base being the most complex organ of the polypide, we laid special focus on its formation and differentiation, but also on its condition in adults.

## Methods

Specimens of *Hislopia malayensis *Annandale, 1916 were collected from the pond of the Faculty of Fisheries of the Kasetsart University in Bangkok (see [13]). Colony pieces were fixed in 1.5 % glutaraldehyde in 0.01 M sodium cacodylate buffer (pH 7.4) for about 1 hour. Specimens were afterwards rinsed three times for 20 minutes in the buffer. Until further preparation in Vienna, specimens were stored in the buffer. Postfixation was conducted with 1% Osmium tetroxide solution in distilled water for 1-2 hours, followed by rinsing in distilled water for about 1 hour. Specimens were afterwards dehydrated with a graded alcohol series prior to embedding the samples into Agar Low-viscosity resin using acetone as intermedium. Eight colony pieces each containing 1-2 buds and several adult zooids were taken for sectioning. Ribbons of serial semi-sections (1 μm thickness) were conducted as described by Ruthensteiner [14]. Sections were stained with toluidine blue and afterwards analysed and photographed with a Nikon DS5M-U1 digital camera mounted on a Nikon Eclipse E800 light microscope. Image stacks from the serial section micrographs were enhanced in contrast, converted to greyscales and imported with an image size of 1024 × 768 into the 3D reconstruction software Amira 4.1 (Mercury Computer Systems, Chelmsford, MA, USA). Alignment of the image stacks was conducted with the AlignSlice tool of Amira. Segmentation of different structures was conducted manually with a brush. A surface for each structure was generated followed by iterated steps of triangle reduction and smoothing (see [14]). Snapshots were taken with the Amira software.

## Results

**Figure 1:**
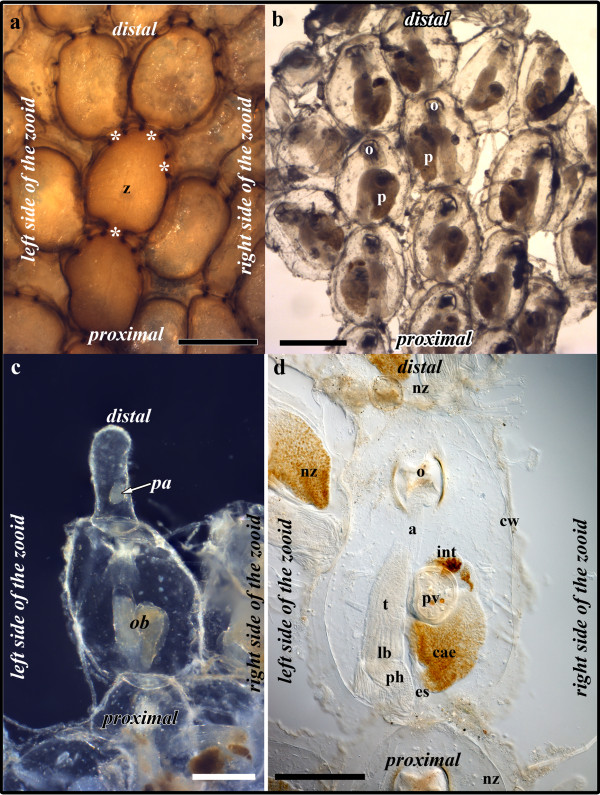
**Overview of *Hislopia malayensis *morphology**. (a) View from the basal side of colony detached from the substrate showing the arrangement of the flat encrusting zooids and the communication sites to neighbouring zooids (asterisks) (on-light, stereo-microscope). (b) Fragment of a colony viewed from the frontal side to show the arrangement of the zooids and the polypides within each zooid. (bright-field transmitted light, stereo-microscope). (c) Older bud already showing the typical oval shape of the adult zooids with a new bud arising as a slender process on its distal side (dark-field transmitted light, stereo-microscope). (d) Detail of a single zooid showing most of the polypides morphological features (differential interference constrast). Abbreviations: a - atrium enclosed by the tentacle sheath, cae - caecum, cw - cystid wall, es - esophagus, int - intestine, lb - lophophoral base, nz - neighbouring zooid, o - orifice, ob - old bud, p - polypide, pa - polypide anlage, ph - pharynx, pv - proventriculus, t - tentacles, z - zooid. Scale bar in (a) and (b) = 400 μm, (c) and (d) = 200 μm.

*Hislopia malayensis *forms flat, encrusting colonies on various substrates (Figure 1.). Each individual zooid is oval-shaped and consists of an outer cystid that protects the polypide which consists of the lophophore carrying the tentacles and the digestive tract (Figure 1b, d). Buds of *H. malayensis *arise on the distal or lateral sides of each zooid throughout the colony (Figure 1a, c). Like in all gymnolaemates, the cystid is formed first during budding and the polypide develops later. In the following selected developmental stages of the development of different organ systems of the polypide will be treated according to their degree of differentiation.

### Stage 1

**Figure 2:**
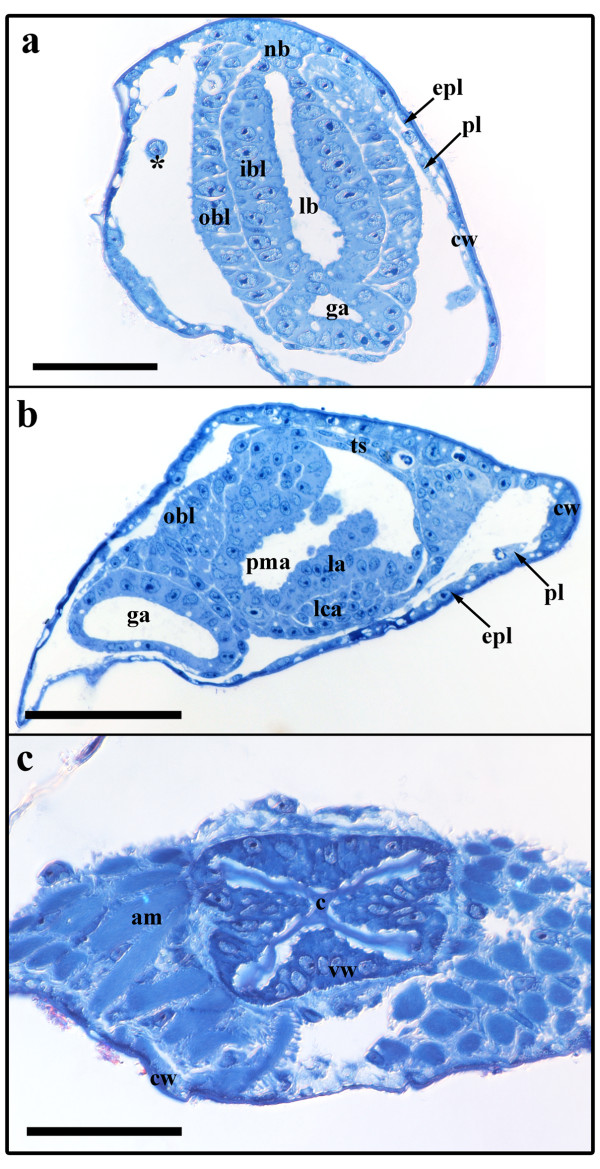
**Sections of early *Hislopia malayensis *buds**. (a) Cross-section through an early bud showing the prominent inner and outer budding layer. Asterisk marks a coelomocyte within the body cavity. (b) Slight oblique section through budding stage 3 showing dense peritoneal cells accumulating at the developing lophophoral base. (c) Cross-section through the vestibular wall of budding stage 5 showing the first signs of collar formation in the vestibulum. Abbreviations: am - apertural muscles, c - developing collar, cw - cystid wall, epl - epidermal layer of the cystid, ga - gut anlage, ibl - inner budding layer, la - lophophore anlage, lb - lumen of the bud, lca - lophophoral coelom anlage, nb - neck of the bud, obl - outer budding layer, pl - peritoneal layer of the cystid, pma - prospective mouth area, ts - tentacle sheath, vw - vestibular wall. Scale bar in (a) and (c) = 30 μm, in (b) 50 μm.

**Figure 3:**
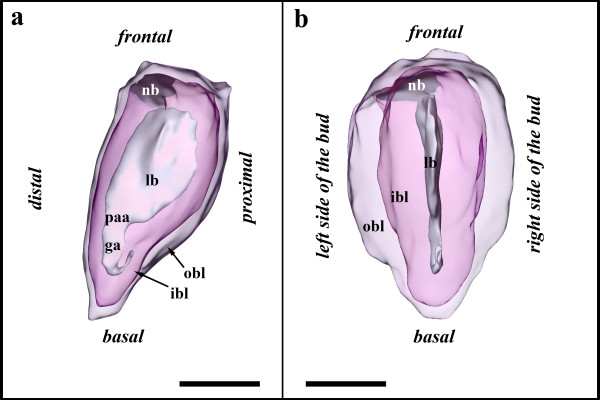
**Budding stage 1 of *Hislopia malayensis***. Inner and outer budding layer displayed transparently. (a) Lateral view of the bud. (b) View on the proximal side of the bud. Abbreviations: ga - gut anlage, ibl - inner budding layer, lb - lumen of the bud, nb - neck of the bud, obl - outer budding layer, paa - prospective anal area. Scale bar = 50 μm.

Early polypide buds arise from the frontal side of the developing cystid. The cystid wall consists of an epidermal and a peritoneal layer (Figure 2a). The epidermal layer of the cystid is easily recognized, whereas the peritoneum is very thin and only sporadically visible on light microscopical level (Figure 2a, b). The early bud probably originates from a proliferation of these layers. However, both of the two involved budding layers, the inner budding layer deriving from the epidermal layer and the outer budding layer from the peritoneum, are prominent and consist of a much thicker epithelium when compared to the remaining cystid wall (Figure 2a). In many instances the peritoneal cells of the cystid resemble coelomocytes that are found floating in the body cavity. It appears that these round, amoeboid coleomocytes originate from the peritoneal epithelium of the cystid (Figure 2a). Budding stage 1 has the shape of a two-layered sac or vesicle that is connected to the cystid frontally via the neck of the bud with the inner layer being continuous with the epidermis and the outer with the peritoneum (Figures 2a; 3a, b). The bud contains a central lumen, bordered by the inner budding layer (Figures 2a; 3a, b). In our stage 1, the lumen is almost club-shaped in its proximo-distal direction, but flat in its lateral dimensions (Figure 3a, b). It extends basally and terminates in a short u-turn (Figure 3a). This extension of the lumen represents the developing gut anlage, which has developed from an invagination from the distally-oriented prospective anal area.

### Stage 2

**Figure 4:**
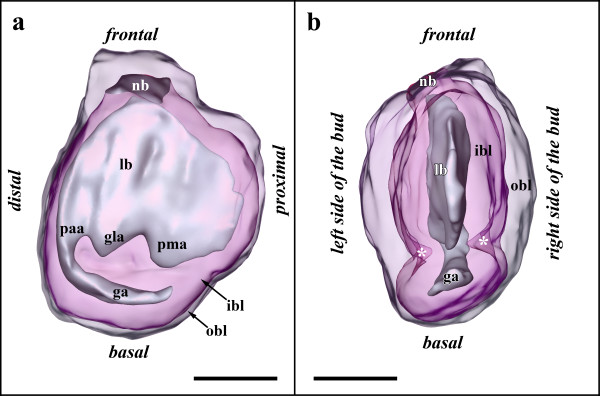
**Budding stage 2 of *Hislopia malayensis***. Inner and outer budding layer displayed transparently. (a) Lateral view of the bud. (b) View on the proximal side of the bud. Asterisks mark the lateral indentations of the budding layers towards the median plane of the bud. Abbreviations: ga - gut anlage, gla - ganglion anlage, ibl - inner budding layer, lb - lumen of the bud, nb - neck of the bud, obl - outer budding layer, paa - prospective anal area, pma - prospective mouth area. Scale bar = 50 μm.

The following budding stage has only slightly altered in its lateral size, but compared to stage 1 is twice as large in the proximo-distal axis. The lumen of the bud has expanded in this axis (Figure 4a). The gut anlage has grown into the proximal direction towards the prospective mouth area on the proximal side of the bud (Figure 4a, b). From the latter a slight indentation indicates the anlage of the prospective mouth area and foregut (Figure 4a). In between the prospective anal and mouth area, the inner budding layer invaginates and forms the anlage of the ganglion, the central nervous system (Figure 4a). The outer budding layer remains comparatively thin, except at both lateral sides of the bud where it pushes in form of two indentations medially into the inner budding layer representing the developing inner peritoneal lining of the gut and the ganglion (Figure 4b).

### Stage 3

**Figure 5:**
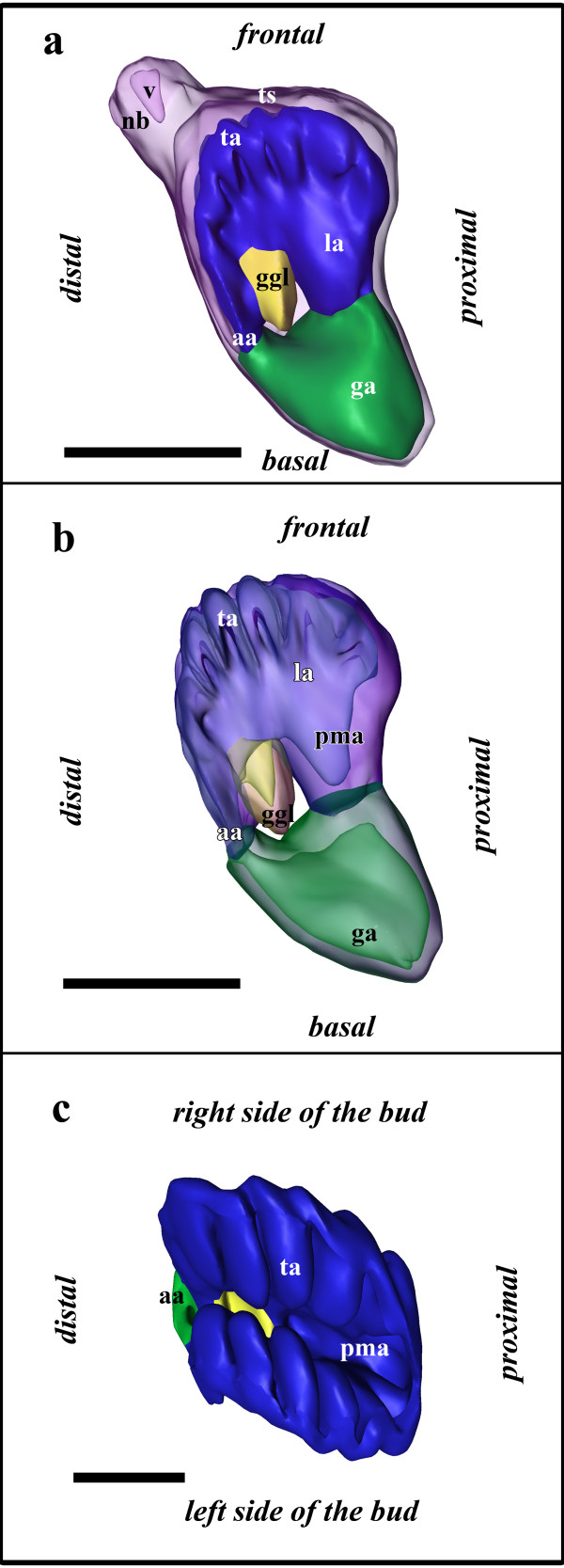
**Budding stage 3 of *Hislopia malayensis***. (a) Lateral view of the bud with the outer budding layer displayed transparently. (b) Similar view as in (a) from the lateral side of the bud with the outer budding layer omitted and the developing gut-, lophophore and ganglion displayed transparently. (c) View from the frontal side of the bud showing the developing tentacles on the lateral lophophoral ridges. Abbreviations: aa - anal area, ga - gut anlage, ggl - ganglion, la - lophophore anlage, nb - neck of the bud, pma - prospective mouth area, ta - tentacle anlagen, ts - tentacle sheath, v - vestibulum. Colors: blue - lophophore anlage, green - gut anlage, purple - outer budding layer, yellow - ganglion anlage. Scale bar in in (a) and (b) = 100 μm, and in (c) = 50 μm.

This budding stage does not show any distinct increase in size, but is characterized by differentiation of the developing organs. Most prominent is the lophophore anlage with the developing tentacles. Two lateral ridges bulge into the lumen of the bud (Figure 5a, b). On each ridge, five tentacle anlagen protrude medially (Figure 5c). Each of them consists of both the inner budding layer (the future tentacle epidermis) and the outer budding layer (the future peritoneal lining). The peritoneal cells are present as a dense mass without any lumen. At the prospective lophophoral base peritoneal cells have aggregated representing the future circum-pharyngeal ring coelom and are wedged in between the prospective foregut and outer peritoneal lining (Figure 2b). On the distal side of the bud, the anal area continues into the anlage of mid- and hindgut (Figure 5a). Compared to budding stage 2, the former now forms a voluminous sac (Figure 5b). On the proximal side of the bud, the anlage of the foregut has advanced from the prospective mouth area towards the anlage of the mid- and hindgut (Figure 5b). In between the prospective mouth and anal area, the ganglion has further invaginated, but is still widely open and in contact with the remaining lumen surrounded by the lophophore anlage (Figure 5b, c). The two lateral indentations of the outer budding layer seen in the previous budding stage have fused medially and form the inner peritoneal lining. As a consequence, the epithelium of the ganglion and the digestive tract are not adjacent anymore (Figure 5b, c). At the neck of the bud on the frontal side, both budding layers have formed the vestibular wall enclosing a small globular cavity, the vestibulum, which is not in communication with the remaining lumen of the bud, i.e. prospective atrium of the zooid (Figure 5a).

### Stage 4

**Figure 6:**
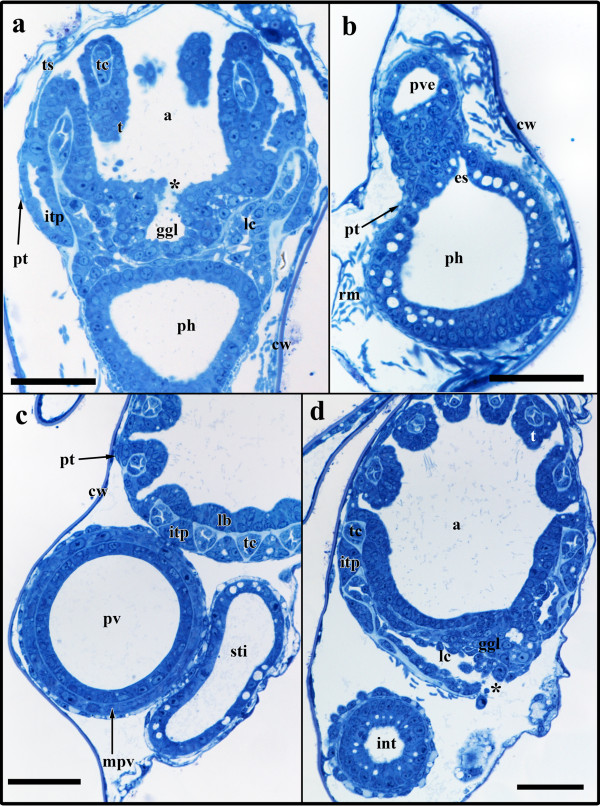
**Sections of *Hislopia malayensis *budding stage 4**. (a) Longitudinal section through the lophophoral base, foregut and ganglion. Asterisk marks the opening of the ganglion towards the atrium/foregut. (b) Section through the short tube-like elongation of the proventriculus to the esophagus. (c) Oblique section through the lophophoral base and parts of the digestive tract. (d) Oblique section through the lophophoral base and ganglion. Asterisk marks the opening of the lophophoral coelom to the remaining body cavity. Abbreviations: a - atrium, cw - cystid wall, es - esophagus, ggl - ganglion, int - intestine, itp - intertentacular pit, lb - lophophoral base, lc - lophophoral coelom, mpv - muscular sheath of the proventriculus, ph - pharynx, pt - peritoneum, pv - proventriculus, pve - tube-like extension of the proventriculus towards the esophagus, rm - retractor muscle fibres, sti - stomach-intestine junction, t - tentacle, tc - tentacle coelom, ts - tentacle sheath. Scale bar = 30 μm

**Figure 7:**
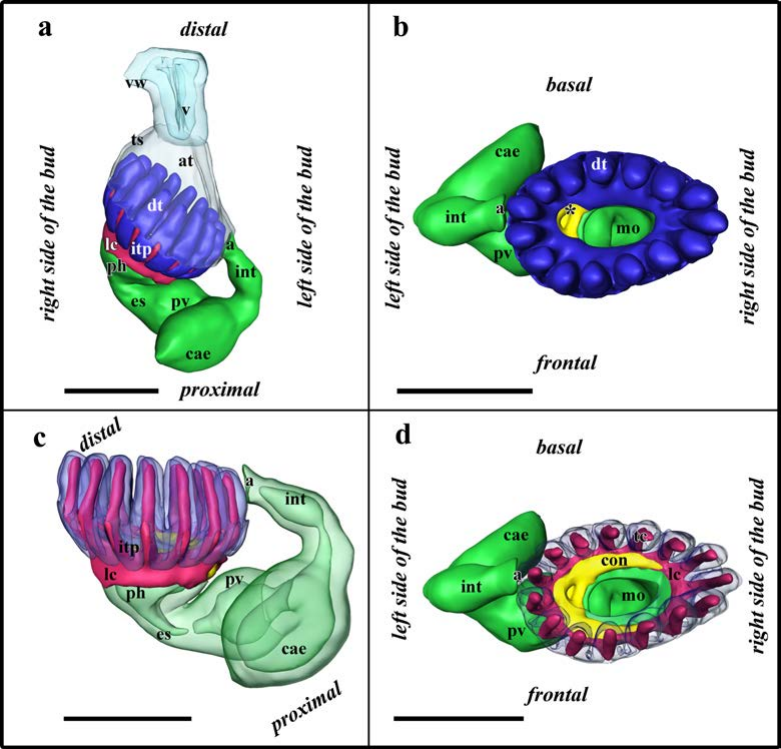
**Budding stage 4 of *Hislopia malayensis***. Because of their thinness, the peritoneal lining of the digestive tract and the lophophoral base were not captured. (a) View from the basal side of the bud. The tentacle sheath and vestibular wall are displayed transparently. (b) View from the distal side of the bud showing the digestive tract as well as the developing lophophore and ganglion. The asterisk marks the opening of the ganglion towards the mouth opening. (c) View from the basal side showing the digestive tract and epidermal layer of the lophophore anlage transparently. (d) View from the distal side showing the epidermal layer of the lophophore anlage transparently. Abbreviations: a - anus, at - atrium, cae - caecum, con - circum-oral nerve trunks, dt - developing tentacles, es - esophagus, int - intestine, itp - intertentacular pits, lc - lophophoral ring coelom, mo - mouth opening, ph - pharynx, pv - proventiculus, ts - tentacle sheath, v - vestibulum, vw - vestibular wall. Colors: blue - lophophore anlage, crimson - lophophoral ring coelom, green - gut anlage, turquoise - vestibular wall, yellow - nervous system. Scale bar = 100 μm.

The developing polypide has bent almost 90° and its longitudinal axis lies in the same plane as the proximo-distal axis of the zooid. The two lateral ridges of the developing lophophore anlage in budding stage 3 have fused on the oral and anal side of the bud and thus form an oval tentacle crown (Figures 6a, d; 7b). The tentacle anlagen, 15 in the analysed stage, are present as small stubs of equal-size projecting distally into the atrium, the space enclosed by the two-layered tentacle sheath (Figures 6a; 7a-c). The latter has approximately doubled in size and both layers have become very thin. Distally the tentacle sheath passes into the vestibular wall, which on the frontal side attaches the bud to the cystid. The vestibulum is x-shaped and is closed towards the exterior and the atrium (Figures 2c; 7a). Proximally, the tentacle sheath terminates at the tentacle bases. In between each pair of tentacles, the tentacle epidermis extends proximally of the tentacle sheath in form of intertentacular pits (Figures 6a, c, d; 7a). Each of these pits contains a hollow canal (Figures 6a; 7c). Medially of the intertentacular pits, the outer budding layer has formed a circum-pharyngeal coelomic compartment, the lophophoral ring coelom (Figures 6a, d; 7a, c). The latter forms an almost complete ring around the pharynx except at the ganglion at the anal side of the lophophoral base where it is confluent with the remaining body cavity (Figure 6d). In between the intertentacular pits the coelom extends from the lophophoral ring into each tentacle. Externally, the lophophoral ring coelom and also the intertentacular pits are covered by a thin peritoneal layer (Figure 6a; because of its thinness not shown in 3D-reconstructions). The ganglion at the lophophoral base has formed two lateral outgrowths that start to form a circum-oral nerve ring (Figure 7d). The ganglion is still in open connection with the mouth/pharyngeal area, but the opening is comparatively smaller than in the previous budding stage (Figures 6a; 7b, d). The digestive tract has for the most part differentiated into the regions found in adult zooids. At the lophophoral base the mouth opening continues into a broad pharynx (Figures 6a; 7a, c). From the latter, the digestive tract continues into a much smaller and still short esophagus (Figures 6b; 7). The esophagus ends blindly and its epithelial wall is in intimate contact with those parts of the digestive tract which derived as an outgrowth from the prospective anal area (Figures 6b; 7c). These parts begin with a short tube-like region which quickly broadens into the bulb-shaped proventriculus (Figures 6c; 7a, c). The proventriculus or cardiac portion of the stomach is thick walled and supplied with a prominent muscular layer in between the peritoneal covering and the epithelium of the digestive tract (Figure 6c). The remaining stomach consists of the caecum. In this budding stage, it has formed a large sac that has bent on the basal side slightly towards the right side of the developing bud (Figure 7a-d). Distally, the digestive tract continues into a short ovoid intenstine which terminates via the anus into the tentacle sheath (Figure 7a-d).

### Stage 5

**Figure 8:**
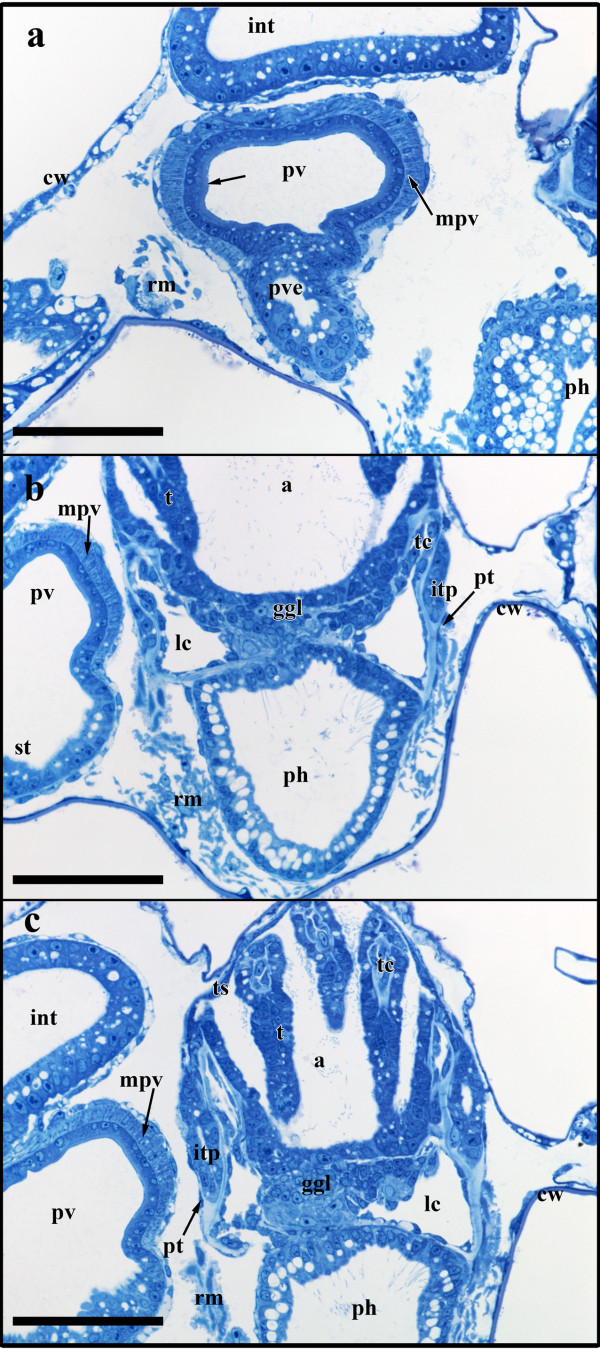
**Sections of *Hislopia malayensis *budding stage 5**. (a) Section through parts of the almost fully differentiated gut in particular the prominent proventriculus. The arrow points to the cuticular inner lining of the latter. (b) Longitudinal section through the lophophoral base and foregut showing the typical cone-shape of the pharynx. (c) Longitudinal section through the lophophoral base and the now compact ganglion. Abbreviations: a - atrium, cw - cystid wall, ggl - ganglion, int - intestine, itp - intertentacular pit, lc - lophophoral coelom, mpv - muscular sheath of the proventriculus, ph - pharynx, pt - peritoneum, pv - proventriculus, pve - tube-like extension of the proventriculus towards the esophagus, rm - retractor muscle fibres, st - stomach, t - tentacle, tc - tentacle coelom, ts - tentacle sheath. Scale bar = 50 μm.

**Figure 9:**
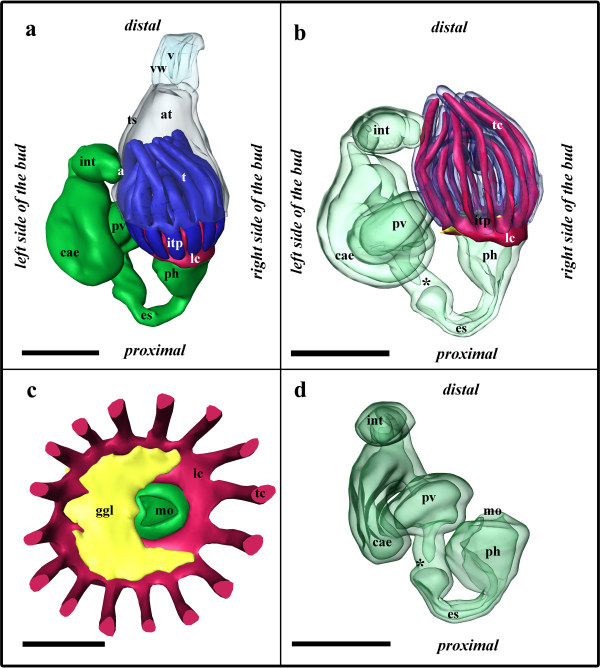
**Budding stage 5 of *Hislopia malayensis***. Because of their thinness, the peritoneal lining of the digestive tract and the lophophoral base were not reconstructed. (a) View from the frontal side of the bud. The tentacle sheath and vestibular wall are displayed transparently. (b) Similar view as in (a) with the tentacle sheath and vestibular wall omitted and the digestive tract and the epidermal layer of the lophophore displayed transparently. Asterisk marks the border of the still separated fore- and mid-gut. (c) Distal view on the lophophoral base showing the lophophoral coelom with the ganglion situated above it and the mouth opening. The surface of the tentacles has been cut. (d) View of the digestive tract displayed transparently. The shape of the pharynx is better visible than in (b). Asterisk marks the border of the still separated fore- and mid-gut. Abbreviations: a - anus, at - atrium, cae - caecum, es - esophagus, ggl - ganglion, int - intestine, itp - intertentacular pits, lc - lophophoral ring coelom, mo - mouth opening, ph - pharynx, pv - proventiculus, t -tentacles, ts - tentacle sheath, v - vestibulum, vw - vestibular wall. Colors: blue - epidermal layer of the lophophore, crimson - lophophoral ring coelom, green - gut anlage, turquoise - vestibular wall, yellow - nervous system. Scale bar in (a), (b) and (d) = 100 μm, in (c) = 50 μm.

The following budding stage is mainly characterised by further differentiation of the different organ systems (Figure 8). The vestibulum has slightly expanded and exhibits no open connection towards the atrium or the exterior (Figure 9a). A collar has started to form within the vestibulum and on sections is present as deeply staining material that is continuous with the ectocyst (Figure 2c). Along with the elongation the tentacle sheath, the tentacles have approximately doubled in their length. At the lophophoral base, the intertentacular pits have grown to measure about 35 μm in length and retain their central canal (Figures 8b, c; 9b, b). The lophophoral ring coelom has not distinctly changed when compared to budding stage 4 and remains confluent with the remaining coelom at the anal side of the ganglion. The latter has closed towards the mouth/pharyngeal area and forms a rather flattened disc distally of the lophophoral ring coelom (Figures 8b, c; 9c). With the exception of the foregut, the digestive tract is characterized by growth and widening of the different regions of the digestive tract, i.e intestine, caecum, proventriculus (Figure 9a, b, d). The pharynx is cone-shaped opening distally with the mouth opening (Figures 8b; 9b, d). Proximally, the esophagus adjoins the pharynx. It has more become elongated and like in the previous budding stage ends blindly towards the cardiac portion of the stomach. At the terminal end it is slightly expanded to the shape of a bulb. From the cardiac portion of the stomach or proventriculus, the thin tube extending to the esophagus has grown longer and is more delimited towards the proventriculus than in the previous budding stage (Figures 8a; 9b, d).

### Condition of the lophophoral base in adult zooids

**Figure 10:**
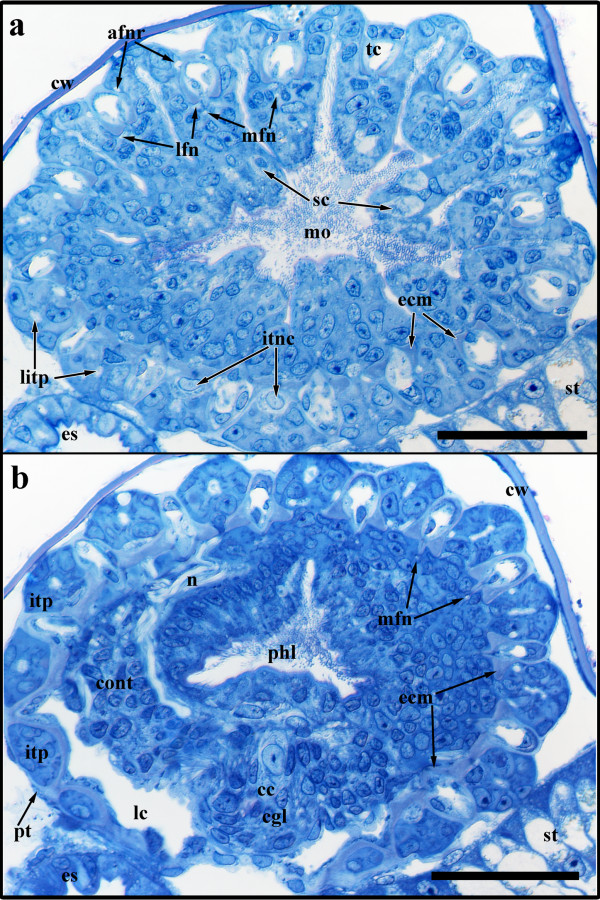
**Sections of the adult lophophoral base of *Hislopia malayensis***. (a) Near-cross-section through the mouth opening distally of the lophophoral base. (b) Near-cross-section through the pharyngeal area more proximally of the lophophoral base than in (a). Abbreviations: afnr - abfrontal nerve root, cc - conspicuous central nerve cell of the cerebral ganglion, cgl - cerebral ganglion, cont - circumoral nerve trunk, cw - cystid wall, ecm - prominently staining extracelluar matrix, es - esophagus, itnc - intertentacular nerve cell, itp - intertentacular pits, lc - lophoral ring coelom, litp - lumen enclosed by the intertentacular pits, lfn - latero-frontal tentacle nerves, mfn - medio-frontal tentacle nerve, mo - mouth opening, n - nerve fibres, phl - pharynx lumen, pt - thin peritoneal layer surrounding the lophophoral base, sc - presumed sensory cells, st - stomach. Scale bar = 30 μm.

**Figure 11:**
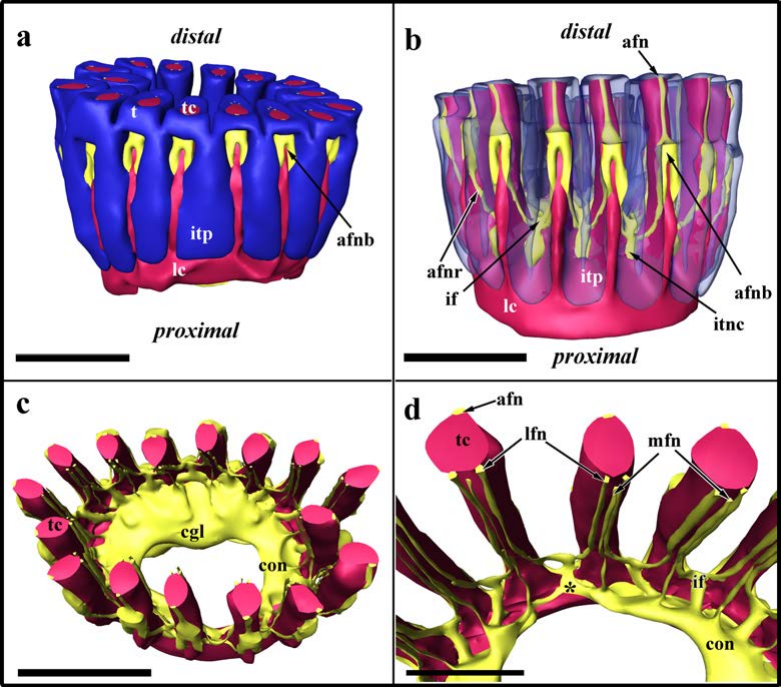
**Adult lophophoral base of *Hislopia malayensis***. (a) Lateral view on the lophophoral base showing its epidermal layer, the lophophoral ring coelom and parts of the nervous system. (b) Similar view as in (a) but with the epidermal layer of the lophophoral base displayed transparently. (c) Slight oblique view from the distal side of the lophophoral base showing the lophophoral coelom and the anally situated central nervous system. (d) Detail of the nervous system showing the junction of the circum-oral nerve ring (asterisk) and the tentacle nerves. Abbreviations: afn - abfrontal tentacle nerve, afnb - abfrontal nerve body, afnr - abfrontal tentacle nerve root, cgl - cerebral ganglion, con - circum-oral nerve trunk, if - intertentacular nerve fork, itnc - intertentacular nerve cell, itp - intertentacular pit, lfn - latero-frontal tentacle nerve, mfn - medio-frontal tentacle nerve, t - tentacle, tc - tentacle coelom Colors: blue - epidermal layer of the lophophoral base, crimson - lophophoral coelom, yellow - nervous system. Scale in (a-c) = 50 μm, in (d) = 20 μm

The adult lophophoral base clearly represents the most complex part of the polypide. The lophophoral ring coelom is similar as in budding stage 5. The intertentacular pits range from 50-60 μm in their length and the epithelium bordering the pits is covered by a weakly staining layer (Figs 10a; 11a, b). In between the intertentacular pits, the tentacle coelom extends from the lophophoral ring coelom into each tentacle (Figure 10a, c). A considerable extracellular matrix (ECM) lies between the epidermal layer and the peritoneum of the tentacles. On light-microscopical cross-sections the medial side of this ECM stains more prominently: More proximally on the lophophoral base it appears either like a zigzag at the tentacle coelom or three-lobed with a pointed median lobe and two large lateral ones which extend towards the median side of the intertentacular pits (Figure 10b). Distally on the lophophoral base only the two lateral lobes are visible as flap-like latero-medial border of the tentacle coelom (Figure 10a).

The nervous system at the lophophoral base forms a circum-oral/pharyngeal nerve ring (Figure 11c, d). The compact central nervous mass, the ganglion, situated at the anal side of the lophophoral base (Figure 11c) contains numerous perikarya and nerve fibres. A single conspicuous perikaryon that is distinctly larger than all remaining nerve cells is situated centrally within the ganglion (Figure 10b). Opposite to the ganglion the circum-oral nerve trunks are connected by a thin bridge (Figure 11d). From the circum-oral nerve ring two principal types of nerves emanate that ultimately innervate the tentacles. The medio-frontal nerve of each tentacle directly emanates in the median plane of each tentacle from the circum-oral nerve ring (Figures 10a, b; 11d). The roots of the latero-frontal and abfrontal tentacle nerves originate from an intertentacular junction which is connected to the circum-oral nerve ring (Figure 11b, d). On the frontal side of the tentacles the medio-frontal tentacle nerves bifurcate from the intertentacular junction and innervate two neighbouring tentacles (Figures 10a; 11d). Similarly, the abfrontal tentacle nerve roots bifurcate on the abfrontal side of the tentacles (Figures 10b, 11a). Proximally of the bifurcation of the abfrontal tentacle nerve roots are conspicuous perikarya that are connected with the intertentacular nervous junction and lie within the wall of each intertentacular pit (Figures 10b, 11a). The abfrontal tentacle nerve roots expand in their diameter along their traverse towards the tentacles. Proximally of the tentacle sheath they fuse into a single abfrontal nerve body (Figure 11a, b). From the latter a single abfrontal tentacle nerve extends into each tentacle (Figure 11b, d). Besides the regular innervations of the tentacles, additional nerve fibres come from the circum-oral nerve ring and innervate cells (probably sensory cells) in the area of the mouth opening. On light microscopical sections, these cells are readily distinguishable by their bright and more translucent cell plasma (Figure 10a).

## Discussion

### Origin of the budding layers

Like in all other bryozoans, the polypide in *H. malayensis *develops from two budding layers; the inner budding layer originates from the epidermis and the outer budding layer from the peritoneum [15]. Some previous investigations described the outer budding layer to form from proliferating epidermal cells [11,16]. More recent observations [17,18] found both layers of the body wall directly involved in the formation of buds. Although this study did not focus on the early bud formation, we never found any peritoneal cells derive from the epidermal layer. In addition, it should be mentioned that the peritoneal layer of the body wall in *H. malayensis *is always inconspicuously thin, even in adult zooids. As a consequence, it is more reasonable to assume the sole thin peritoneal layer to form the outer budding layer. Whether coelomocytes liberated from the peritoneal layer, as observed in the current study in early buds, participate in the formation of the outer budding layer remains unanswered. Different kinds of coelomocytes within the body cavity have been reported in representatives of all bryozoan clades. In adult zooids, they possibly act in phagocytosis of excretory substances [1]. Their role during budding could be similar in accumulation of metabolic waste created during budding. On the other hand, coelomocytes may be involved in the formation of peritoneally derived tissues, such as muscles. A similar function has been indicated for phylactolaemate coelomocytes [9].

### Formation of the lophophore

The initial lophophore anlage develops as two lateral ridges in *H. malayensis*. A similar formation has been described for all other bryozoan clades (Cyclostomata: [19], Cheilostomata: e.g. [18,20], Ctenostomata: [10,11], Phylactolaemata: [9]). In *Paludicella articulata *the lateral ridges first unite at the oral side, while the tentacles on the anal side are the last to form [10]. In the current study on *H. malayensis*, a stage showing a U-shaped arrangement of the developing tentacles was not encountered. However, in our budding stage 3 of *H. malayensis*, the lophophoral ridges bulge slightly inward on the oral side, whereas they abruptly end on the anal side as described in *P. articulata*. A similar formation of the lophophore is described for the cheilostome *Membranipora membranacea *[20] and the phylactolaemate *Cristatella mucedo *[9]. The Phylactolaemata, however, show some differences regarding the formation of the lophophore which are also reflected in their adult condition. The lateral lophophoral ridges or more precisely bulges form the large lophophoral arms giving this clade the typical horse-shoe shaped lophophore. At first these do not carry tentacles in phylactolaemates [9] as seen in members of the Ctenostomata [10] and the Cheilostomata [18,20]. In contrast to the remaining, pre-dominantly marine clades, the oral tentacles are the first to be formed in the Phylactolaemata [9,21,22]. However, these differences are again reflected in the condition of the coelomic compartments of the adults. In the Phylactolaemata the ring-canal on the oral side of the lophophore base is comparatively short supplying only few tentacles [9], whereas the ring canal in the Ctenostomata (*H. malayensis*, this study) and Cheilostomata (*Cryptosula pallasiana*, [23]) encompasses almost the entire lophophoral base. Accordingly, two major patterns in the development of the lophophore can be recognized from the currently available data: 1. it starts with paired lateral anlagen that first close on the oral side and later also on the anal side and 2. the first tentacles arise on the area of the prospective ring canal with the most medial ones on the oral side to appear last.

### Intertentacular pits of the lophophoral base

As stated by Gordon [23], the lophophoral base represents the most complex structure of the bryozoan polypide. Surprisingly, his detailed study of the organization in the cheilostome *Cryptosula pallasiana *currently remains the only study for bryozoans. In *H. malayensis *we describe intertentacular pits at the lophophoral base for the first time in a ctenostome. A similar, most probably homologous structure is present in *C. pallasiana*. In the latter they are called ciliated pits and measure 25 - 30 μm in length [1]. In adult specimens of *H. malayensis *they are approximately two times longer than in *C. pallasiana *and consequently much more noticeable. In *C. pallasiana *the pits are covered by a cuticle and the lining cells possess cilia projecting into the lumen of the pit. In *H. malayensis *a thin acellular layer, most likely cuticle, lines the intertentacular pits as well, but the confirmation of the presence of cilia would require electron microscopic examination. Conspicuous intertentacular pits also occur in several other ctenostome bryozoans (Schwaha, unpublished data). Consequently, it seems likely to expect them in more cheilostomes as well and thus might be a synapomorphy for these two clades. As in *C. pallasiana *[23], we currently can give no indication about the function of the intertentacular pits.

### Formation of the central nervous system and adult condition

In *H. malayensis *the central nervous system forms by an invagination of the inner budding layer (epidermal layer) in between the prospective mouth and anus, thus being identical to all other bryozoan classes in this respect [8,9]. However, in contrast to the Phylactolaemata, the ganglion in *H. malayensis *never contains a lumen and thus is compact in late budding stages (stage 5) and adults.

The nervous system, in particular at the lophophoral base and the tentacle innervation, has been subject of several studies in phylactolaemates and gymnolaemates [1,23-31]. So far, the central nervous system in cyclostome bryozoans remains unstudied. Tentacle innervation, however, is briefly mentioned by Nielsen & Riisgard [32]. All studied bryozoans possess a circum-oral/pharyngeal nerve ring. Tentacles are innervated by 4-6 nerves. Four of these tentacle nerves (the abfrontal, frontal and the paired latero-frontal nerves) are located subepidermally, while the remaining two are located subperitoneally. Only the phylactolaemate *Asajirella gelatinosa *shows a slightly different configuration of the subepidermal nerves [1]. In the current study on *H. malayensis*, we were only able to locate the four subepidermal nerves and not the paired subperitoneal ones. Only in the cheilostome *Cryptosula pallasiana*, the full set of six tentacle nerves was detected [23]. In the cyclostome *Crisia eburnea *[32] and the cheilostome *Electra pilosa *[33] the four subepidermal nerves were confirmed, whereas only the two subperitoneal and the latero-frontal tentacle nerves were found in *Flustrellidra hispida, Membranipora membranacea *[28], *Farrella repens *and *Alcyonidium *sp. [29]. In phylactolaemate bryozoans radial nerves extend from the nerve ring in between the tentacles, in the intertentacular membrane. Towards the tentacles, the radial nerves bifurcate within the intertentacular membrane and branch off the tentacles nerves [1,27]. This intertentacular origin of the tentacles nerves resembles the abfrontal and latero-frontal tentacle nerves of *H. malayensis*. However, the medio-frontal tentacle nerves branch off directly from the circum-oral nerve ring in *H. malayensis*. In the ctenostomes *Flustrellidra hispida *[28], *Alcyonidium *sp., *Farrella repens *[29] and the cheilostomes *Membranipora membranacea *[28] and *Electra pilosa *[33] only one pair of tentacle nerves were found to originate from an intertentacular origin, i.e. the latero-frontal nerves. In *E. pilosa *the abfrontal nerve extends directly from the circum-oral nerve ring, whereas it was not detected at all in the other aforementioned species - most likely a result of methodological problems of vital staining of nervous tissue. Nonetheless, summing up the little information available the following trend seems to be present in bryozoans: In the Phylactolaemata all tentacle nerves are of intertentacular origin, while gymnolaemates subsequently branch off tentacle nerves directly from the nerve ring, first the medio-frontal nerve (Ctenostomata: *H. malayensis*, this study) and then the abfrontal nerve as well (Cheilostomata: *E. pilosa *[33]). This trend coincides with current opinions of bryozoan phylogeny, with the Phylactolaemata as most basal branch and the Ctenostomata being paraphyletic as ancestors of the Cheilostomata. However, a broader range of taxa (including the neglected Cyclostomata) need to be studied to confirm this trend. Additionally, the basic number of tentacle nerves needs to be clarified. In several Phylactolaemata [1,34] and the cheilostome *E. pilosa *[33], 'subperitoneal' or 'enclosed' peritoneal cells that are topologically identical to the position of the subperitoneal nerves described for *C. pallasiana *[23] and several ctenostomes [28,29] were described. Consequently, it seems probable that these cells represent nerves and that they can be expected in most if not all bryozoans, also in *H. malayensis*, but their detection requires detailed electron microscopic or state-of-the-art immunocytochemical studies.

### Formation of the gut and the esophagus-cardia length

The mid- and hindgut in *H. malayensis *form as anal outpocketing as described for the ctenostomes *Flustrellidra hispida *[35], *Paludicella articulata *[10] and *Pottsiella erecta *[36]. As recently summarized by Schwaha et al. [9], diverging descriptions of gut formation have been provided. Some authors claim an oral outpocketing to give rise to these parts of the digestive tract [11,37]. Considering the similarities in the formation of all other organ systems during budding of bryozoans, it appears more probable that the mid- and hindgut of bryzoans generally develop from an anal outpocketing. Ultimately, reinvestigating and increasing the number of species in all clades needs to be conducted to confirm this suggestion.

As mentioned by Rogick [38], the gut terminology of bryozoans is in a 'nice state of confusion'. Only Silen [39] attempted to give a general terminology to the various parts of the digestive tract by considering all bryozoan classes. Two valve-like constrictions, one at the end of the foregut and a second before the intestine (or rectum), are important criteria for assigning terms for specific gut regions [39]. The valve at the end of the foregut is commonly termed cardiac valve or esophageal valve and represents the border between the esophagus and the cardia. As seen in the current study on *H. malayensis*, previous studies on the Phylactolaemata [9] as well as the Cyclostomata [19] this valve develops at the border of the two anlagen assembling the gut during budding. In the Phylactolaemata and the Stenolaemata the digestive tract from the caecum to the mouth opening is short, while it is usually elongated in gymnolaemates. Based on the distal position of the cardiac valve in the latter, this elongated tube was considered to be a result of the elongation of the cardia. Consequently, an esophagus was stated to be absent in gymnolaemates, because no proper differentiation towards the pharynx is given [39]. While this might be true for several cheilostome species (e.g. *Membranipora*: [39]; *Bugula: *[16]; *Cryptosula*: [16,40]; *Electra*: [16]; *Hippothoa*: [41]; *Lageneschara*: [42], also see [1,43]), ctenostome bryozoans show a larger variation concerning this feature of the gut. Our results on *H. malayensis *show that the cardiac valve is situated far proximally and most of the tube-like elongation develops and consists of the foregut, the esophagus. Only a comparatively small part of the tube is composed of the cardia distally of the muscular proventriculus. An identical arrangement is present in the hislopiids *H. corderoi *[44] and *Echinella placoides *[45]. In contrast, in the only ctenostome superfamily showing similar flattened box-shaped zooids, the Alcyonidioidea, the esophagus is negligibly small and the cardiac tube elongated [46,47]. An elongated esophagus is generally considered to be present in 'stoloniferan' ctenostomes [48] in which the polypide bud becomes dislocated into an elongated peristome that later is separated from the remaining 'stolon' [49,50]. However, in other ctenostomes with elongated peristomes but lacking true stolons, like the Victorellidae, the esophagus and cardia are both present as almost equally long tubes. In addition, the relative size, particularly of the cardiac tube, is affected by the state of its contraction [51]. It seems worthwhile to investigate whether the differences in the morphology of the gut prove to be valuable for drawing phylogenetic inferences. Comparative data is currently sparse, because the location of the cardiac valve is only given for very few species. Since the cardiac valve hinders reflux of food particles from the cardiac stomach, it seems more reasonable that the anatomy of the gut is influenced by the diet and the mode of digestion.

## Conclusions

Compared with the recent study of the phylactolaemate *Cristatella mucedo *[9] and older studies, we are able to show that the development of the polypide shows distinct similarities in the formation of the different organ systems. These include the early polypide bud formation as a proliferation of epidermal cells bulging towards the peritoneal layer of the bud, a two-layered vesicle-like early bud, the central nervous system or ganglion forming as an invagination of the epidermal layer in between the prospective mouth and anal area, the digestive tract mainly forming from an outpocketing of the prospective anal area that grows towards a comparatively small anlage of the foregut (pharynx and esophagus), and the lophophore forming from two lateral anlagen that first fuse on the oral and afterwards on the anal side. These similarities found between phylactolaemates and ctenostomes thus support the monophyly of Bryozoa.

The site where the anlage of the mid/hind-gut and the foregut meet is represented in adult zooids by the cardiac valve. A comparison of different bryozoan species and superfamilies shows that its location is not identical in gymnolaemates which always possess an elongated tube-shaped gut connecting the pharynx with the caecum. With the current paucity of comparative data, it is more appropriate to consider the diet and the mode of digestion to be decisive on the variable location of the cardiac valve.

At the complex lophophoral base of adult zooids intertentacular pits of unknown function are described for the first time in a ctenostome. Similar structures were only reported in the cheilostome *C. pallasiana *[23]. It is likely that they are present in more if not all gymnolaemate species, but have escaped the attention of previous investigators. Along with structures of the nervous system at the lophophoral base and the tentacle innervation, these characters appear promising for further analysis for comparative phylogenetic purposes on bryozoans.

With the polypide development of the Phylactolaemata [9] and Ctenostomata (this study) studied in more detail with modern visualisation techniques, the Cyclostomata remain an essential taxon for further study. Organogenesis in the budding process of the later was only studied by Borg [19] and Nielsen [37], but is only poorly documented. In cyclostome bryozoans the polypide is formed first and the cystid later. This formation of buds is also found in the basal Phylactolaemata, in contrast to budding of the Cteno- and Cheilostomata where the cystid is formed first and the polypide later. Accordingly, future studies should concentrate on cyclostome budding to complement our knowledge on developmental patterns of bryozoans.

## Competing interests

The authors declare that they have no competing interests.

## Authors' contributions

TS conducted all practical work and wrote the manuscript. TW coordinated research in Thailand, collected and identified the animals and contributed significantly to the manuscript. All authors read and approved the final version of the manuscript.
